# ﻿Three new species of *Grandilithus* Liu & Li, 2022 (Araneae, Phrurolithidae) from southern China

**DOI:** 10.3897/zookeys.1183.113075

**Published:** 2023-11-07

**Authors:** Mengjiao Xu, Yannan Mu, Chao Zhang, Feng Zhang

**Affiliations:** 1 Key Laboratory of Zoological Systematics and Application of Hebei Province, Institute of Life Science and Green Development, College of Life Sciences, Hebei University, Baoding, Hebei 071002, China Hebei University Baoding China

**Keywords:** Diagnosis, morphology, spider, taxonomy

## Abstract

Three new species of *Grandilithus* Liu & Li, 2022 are described from southern China on the basis of both sexes: *G.chongzuo***sp. nov.** from Guangxi, *G.xiaohuangshan***sp. nov.** from Guangdong and *G.lanxi***sp. nov.** from Jiangxi. A distribution map of these three species is provided.

## ﻿Introduction

Phrurolithidae Banks, 1892 is a spider family comprising 26 genera and 388 species from most parts of the world, with 17 genera and 202 species reported from China (WSC 2023). Among them, seven newly established and three newly recorded genera have been reported from China in recent years (Liu et al. 2020; Jin et al. 2022; Liu et al. 2022; Mu and Zhang 2022; Mu et al. 2022; Mu and Zhang 2023a, 2023b).

The genus *Grandilithus* Liu & Li, 2022 was established based on the type species *G.anyuan* Liu & Li, 2022. There are 29 species known from China, and two each have been reported from Vietnam and Japan (WSC 2023). Species of *Grandilithus* can be recognized by the straight posterior eye row and the narrow dorsal scutum less than 1/2 of the abdomen length. Males of this genus can be distinguished by the presence of a strong, well-developed extension on the distal palpal femur (femoral apophysis), a small tubercle on the ventral tibia, a retrolateral tibial apophysis with a curved tip, the thin embolus curved as a semicircle, and the absence of a conductor. The females of this genus can be distinguished by the broad median septum and the elongated spermathecae (Liu et al. 2022).

While examining specimens from southern China, three new *Grandilithus* species were discovered, which are described in this paper.

## ﻿Materials and methods

All measurements are given in millimeters. The leg measurements are shown as: total length (femur, patella, tibia, metatarsus, tarsus). The epigynes were removed and cleared in a pancreatin solution (Álvarez-Padilla and Hormiga 2007) and then transferred to 95% ethanol. All specimens are preserved in 95% alcohol. Photographs were taken using the Leica M205A stereomicroscope equipped with a DFC 550 CCD. Final figures were retouched using Adobe Photoshop. All specimens are deposited in the Museum of Hebei University (MHBU), Baoding, China.

The following abbreviations are used: AER, anterior eye row; ALE, anterior lateral eye; AME, anterior median eye; CH, clypeal height; CRW, cephalic region width; CW, carapace width; EAW, eye area width; MOA, median ocular area; PLE, posterior lateral eye; PME, posterior median eye; Spination: d, dorsal; pl, prolateral; pv, proventral; rv, retroventral.

## ﻿Taxonomy

**Family Phrurolithidae Banks, 1892**

### ﻿Genus Grandilithus

Taxon classificationAnimaliaAraneaePhrurolithidae

Liu & Li, 2022

2B15B68A-50BE-583C-8B87-68B9A6D23C88

#### Type species.

*Grandilithusanyuan* Liu & Li, 2022.

### ﻿ Grandilithus chongzuo sp. nov.

Taxon classificationAnimaliaAraneaePhrurolithidae

E4CFEE91-141C-564B-B0C4-4E40D69086CE

https://zoobank.org/DAFF490C-B867-47EB-95B2-7A8A19381A3F

**Figure 1.**
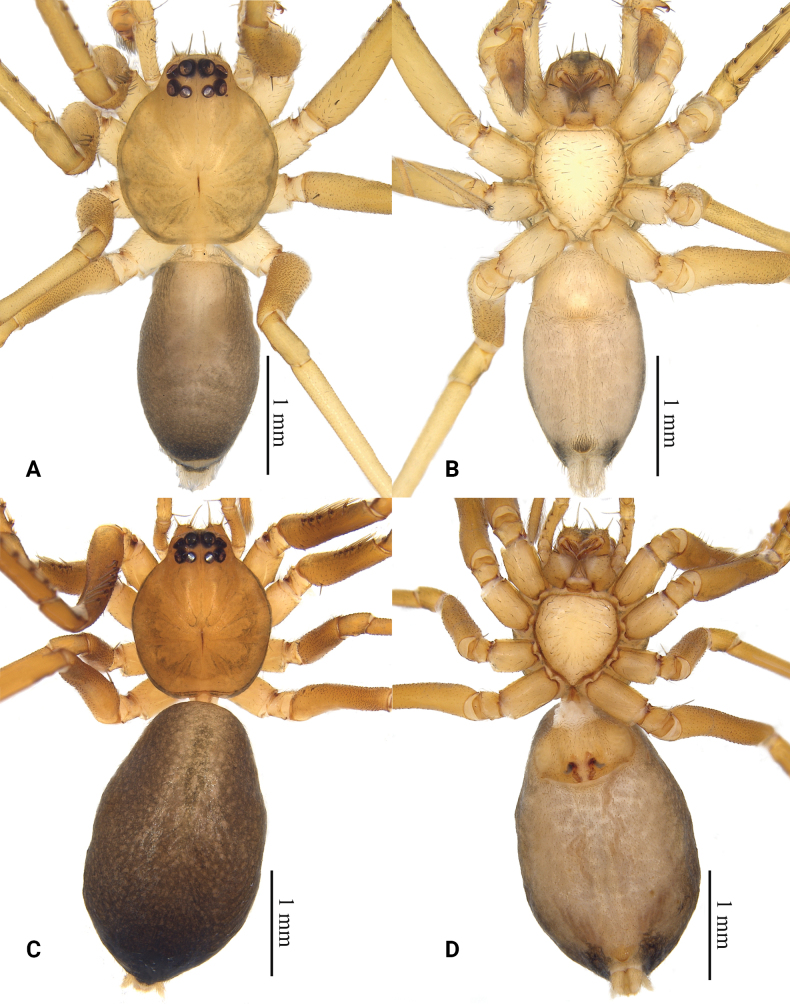
Habitus of *Grandilithuschongzuo* sp. nov. **A** male holotype, dorsal view **B** same, ventral view **C** female paratype, dorsal view **D** same, ventral view.

**Figure 2.**
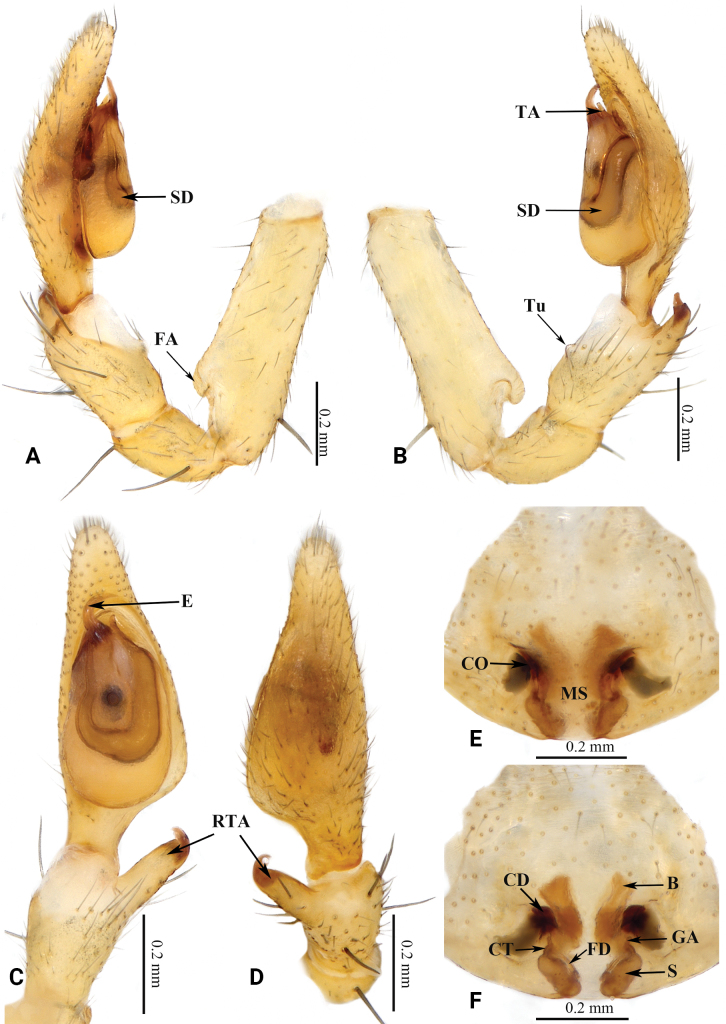
Copulatory organs of *Grandilithuschongzuo* sp. nov. **A** male left palp, prolateral view **B** same, retrolateral view **C** same, ventral view **D** same, dorsal view **E** epigyne, ventral view **F** same, dorsal view. Abbreviations: B—bursa; CD—copulatory duct; CO—copulatory opening; CT—connecting tube; E—embolus; FA—femoral apophysis; FD—fertilization duct; GA—glandular appendage; MS—median septum; RTA—retrolateral tibial apophysis; S—spermatheca; SD—sperm duct; TA—tegular apophysis; Tu—tubercle.

Figs 1 , 2 Chinese name: 崇左大斑蛛

#### Type material.

***Holotype*** ♂ (GXCZ-16-46): China: Pairu Village, Zuozhou Town, Chongzuo City, Guangxi Zhuang Autonomous Region (22°34.40'N, 107°25.36'E; 203 m a.s.l.), 4 November 2016, leg. Guiqiang Huang. ***Paratype***: 2♀, with same data as holotype.

#### Etymology.

The specific epithet refers to the type locality.

#### Diagnosis.

This species resembles *G.nonggang* (Liu, Xu, Xiao, Yin & Peng, 2019) in having a similarly shaped embolus and sperm duct, but can be recognized by: 1) the long RTA, with thin base and coracoid-shaped tip (vs RTA short, base wide, cf. Fig. 2B–D with fig. 11B–D in Liu et al. 2019); 2) the oval tegulum and the slightly curved tegular apophysis without an expanded tip (vs tegulum nearly circular, tegular apophysis straight with an expanded tip, cf. Fig. 2B–D with fig. 11C in Liu et al. 2019); 3) the short copulatory duct (vs long, cf. Fig. 2E, F with fig. 12B, C in Liu et al. 2019); 4) the small, thin glandular appendage (vs thick, cf. Fig. 2E, F with fig. 12B, C in Liu et al. 2019); and 5) the small spermathecae, with thin connecting tubes (vs connecting tubes strong and thick, spermathecae large, cf. Fig. 2E, F with fig. 12B, C in Liu et al. 2019).

#### Description.

**Male (holotype)**: total length 3.27, carapace 1.56 long, 1.34 wide; abdomen 1.71 long, 1.00 wide. Eye sizes and interdistances: AME 0.14, ALE 0.12, PME 0.09, PLE 0.11; AME–AME 0.05, AME–ALE 0.02, ALE–ALE 0.33, PME–PME 0.10, PME–PLE 0.04, PLE–PLE 0.37, ALE–PLE 0.08. EAW 0.52, CRW 0.68, EAW/CRW 0.76, CRW/CW 0.51. MOA 0.34 long, anterior width 0.32, posterior width 0.29. CH 0.13. CH/AME 0.93. Labium 0.14 long, 0.23 wide. Sternum 0.89 long, 0.77 wide. Leg measurements: I 8.41 (2.07, 0.56, 2.36, 1.92, 1.50), II 6.58 (1.73, 0.51, 1.70, 1.44, 1.20), III 5.42 (1.44, 0.47, 1.16, 1.42, 0.93), IV 8.79 (2.37, 0.57, 2.04, 2.44, 1.37), leg pattern: 4123. Spination: femur I–IV d 1, femur I pl 5, femur II pl 3, tibia I pv 9 rv 9, tibia II pv 9 rv 8, metatarsus I pv 4 rv 4, metatarsus II pv 4 rv 3.

***Coloration*.** Carapace yellowish, with several patches resembling flowing droplets beside fovea. Abdomen gray, with a small, fawn dorsal scutum anteriorly and several lighter colored transverse stripes posteriorly. A small, irregular, slightly sclerotized area in front of the spinnerets with black setae. Leg light yellow.

***Palp*** as in Fig. 2A–D. Femoral apophysis protruding. Tubercle distinct. Retrolateral tibial apophysis long, tip curved, coracoid-shaped. Tegulum oval, thinner than cymbium; tegular apophysis slightly curved, tip not expanded. Sperm duct distinct, tapering from retrolateral of tegulum to base of embolus. Embolus curved, tip sharp.

**Female (paratype)**: total length 4.18, carapace 1.51 long, 1.30 wide; abdomen 2.67 long, 1.61 wide. Eye sizes and interdistances: AME 0.13, ALE 0.12, PME 0.08, PLE 0.10; AME–AME 0.05, AME–ALE 0.01, ALE–ALE 0.32, PME–PME 0.09, PME–PLE 0.04, PLE–PLE 0.35, ALE–PLE 0.06. EAW 0.51, CRW 0.66, EAW/CRW 0.77, CRW/CW 0.51. MOA 0.30 long, anterior width 0.30, posterior width 0.27. CH 0.11, CH/AME 0.85. Labium 0.13 long, 0.24 wide. Sternum 0.85 long, 0.77 wide. Leg measurements: I 7.75 (1.94, 0.56, 2.30, 1.70, 1.25), II 6.15 (1.55, 0.48, 1.77, 1.23, 1.12), III 4.99 (1.32, 0.45, 1.11, 1.32, 0.79), IV 7.97 (2.15, 0.54, 1.86, 2.27, 1.15), leg pattern: 4123. Spination: femur I–IV d 1, femur I pl 6, femur II pl 4, tibia I pv 10 rv 10, tibia II pv 9 rv 9, metatarsus I pv 4 rv 4, metatarsus II pv 4 rv 3.

***Coloration*.** Carapace yellow-brown, darker than male, with several patches resembling flowing droplets beside fovea. Abdomen dark gray without dorsal scutum. A small, irregular, slightly sclerotized area in front of the spinnerets with black setae. Leg yellow-brown.

***Epigyne*** as in Fig. 2E, F. Epigynal plate slightly sclerotized. Median septum broad. Copulatory openings small, separated by median septum. Copulatory ducts bent. Bursa nearly rectangular. Glandular appendages thin, short, cylindrical. Connecting tubes curved, thinner than copulatory ducts. Spermathecae small, oval, slanted, separated. Fertilization ducts located at posterior of spermathecae.

#### Distribution.

China: Guangxi Zhuang Autonomous Region (Fig. 7).

### ﻿ Grandilithus xiaohuangshan sp. nov.

Taxon classificationAnimaliaAraneaePhrurolithidae

A59E6777-5879-5BF2-9114-B8665BAC28CE

https://zoobank.org/F7AB13E6-C2BD-4AB5-8CDF-BEF871120A06

**Figure 3.**
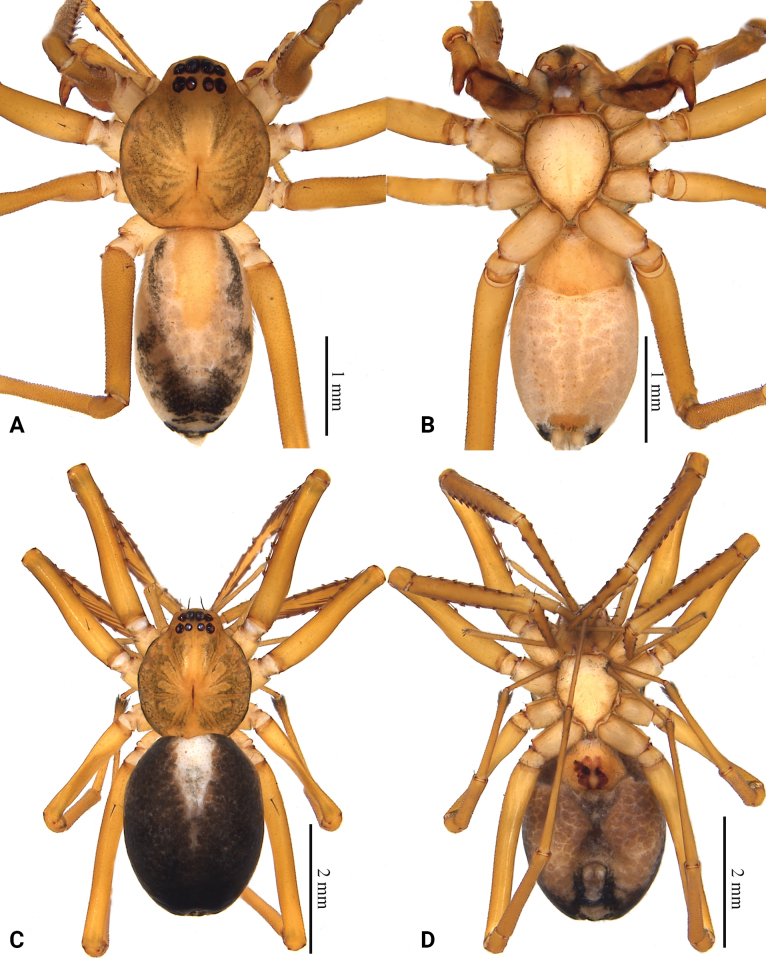
Habitus of *Grandilithusxiaohuangshan* sp. nov. **A** male holotype, dorsal view **B** same, ventral view **C** female paratype, dorsal view **D** same, ventral view.

**Figure 4.**
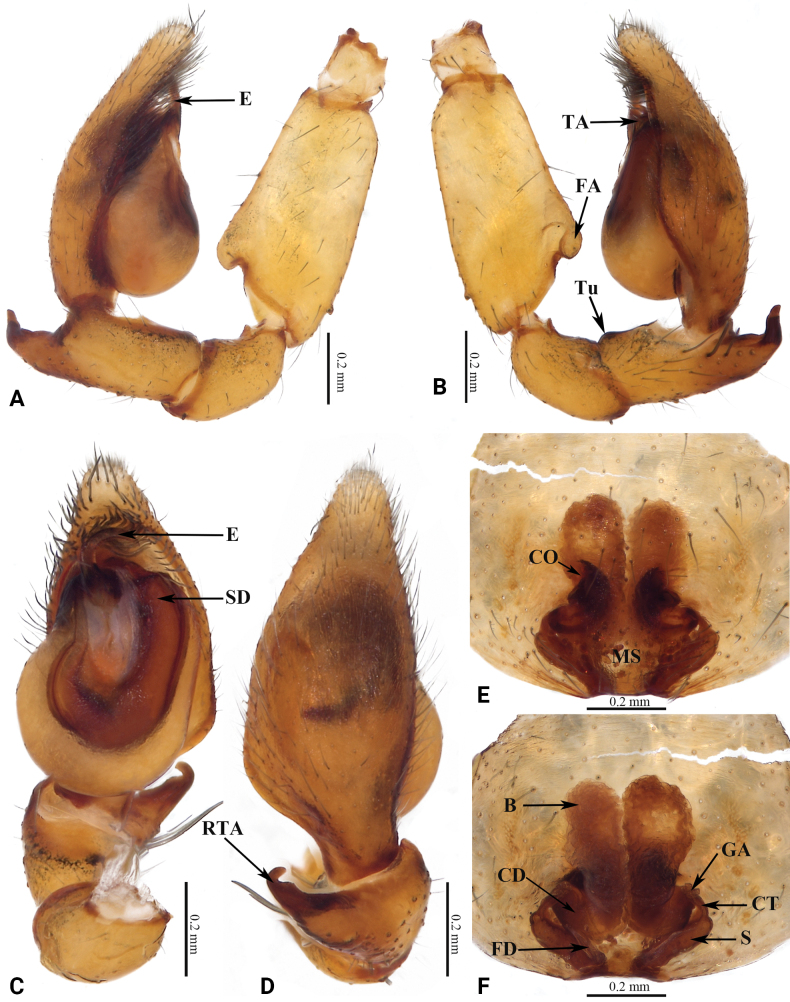
Copulatory organs of *Grandilithusxiaohuangshan* sp. nov. **A** male left palp, prolateral view **B** same, retrolateral view **C** same, ventral view **D** same, dorsal view **E** epigyne, ventral view **F** same, dorsal view. Abbreviations: B—bursa; CD—copulatory duct; CO—copulatory opening; CT—connecting tube; E—embolus; FA—femoral apophysis; FD—fertilization duct; GA—glandular appendage; MS—median septum; RTA—retrolateral tibial apophysis; S—spermatheca; SD—sperm duct; TA—tegular apophysis; Tu—tubercle.

Figs 3 , 4 Chinese name: 小黄山大斑蛛

#### Type material.

***Holotype*** ♂ (HBUARA#2021-63): China: Xiaohuangshan Scenic Spot, the Nanling Mountain National Forest Park, Ruyuan County, Shaoguan City, Guangdong Province (24°53.72'N, 113°1.24'E; 1338 m a.s.l.), 23 May 2021, leg. Yannan Mu. ***Paratype***: 2♀, with same data as holotype.

#### Etymology.

The specific epithet refers to the type locality.

#### Diagnosis.

This new species resembles *G.florifer* (Fu, He & Zhang, 2015) in having a similarly shaped embolus, but can be recognized by: 1) the different color pattern and lighter color (vs darker color, cf. Fig. 3 with figs 21, 22 in Fu et al. 2015); 2) the broad tegulum (wider than cymbium), the small, arch-shaped tegular apophysis (vs tegulum thinner than cymbium, tegular apophysis long, straight, cf. Fig. 4B, C with figs 25, 26 in Fu et al. 2015); 3) the thin median septum (vs wide, cf. Fig. 4E with figs 27, 28 in Fu et al. 2015); 4) the thick and strong copulatory ducts (vs thin and small, cf. Fig. 4E, F with figs 27, 28 in Fu et al. 2015); and 5) the long, straight spermathecae, with thin connecting tubes (vs connecting tubes thick, spermathecae short and curved, cf. Fig. 4F with figs 27, 28 in Fu et al. 2015).

#### Description.

**Male (holotype)**: total length 3.70, carapace 1.67 long, 1.48 wide; abdomen 2.03 long, 1.18 wide. Eye sizes and interdistances: AME 0.12, ALE 0.10, PME 0.08, PLE 0.09; AME–AME 0.06, AME–ALE 0.01, ALE–ALE 0.32, PME–PME 0.10, PME–PLE 0.06, PLE–PLE 0.40, ALE–PLE 0.10. EAW 0.54, CRW 0.75, EAW/CRW 0.72, CRW/CW 0.51. MOA 0.29 long, anterior width 0.30, posterior width 0.27. CH 0.12. CH/AME 1.00. Labium 0.16 long, 0.24 wide. Sternum 0.98 long, 0.80 wide. Leg measurements: I 9.10 (2.14, 0.62, 2.66, 2.20, 1.48), II 7.14 (1.82, 0.57, 1.90, 1.68, 1.17), III 5.95 (1.57, 0.49, 1.34, 1.58, 0.97), IV 8.94 (2.41, 0.56, 2.19, 2.49, 1.29), leg pattern: 1423. Spination: femur II–IV d 1, femur I pl 5, femur II pl 3, tibia I pv 8 rv 9, tibia II pv 8 rv 8, metatarsus I pv 4 rv 4, metatarsus II pv 4 rv 3.

***Coloration*.** Carapace yellow-brown, with several patches resembling flowing droplets beside fovea. Abdomen fawn, with a small, yellow dorsal scutum anteriorly and black pattern posteriorly. A small, irregular, slightly sclerotized area in front of the spinnerets with black setae. Leg yellow-brown.

***Palp*** as in Fig. 4A–D. Femoral apophysis protruding. Tubercle indistinct. Retrolateral tibial apophysis with broad base, curved, with coracoid-shaped tip. Tegulum nearly circular, wider than cymbium; tegular apophysis small. Sperm duct distinct, tapering from retrolateral of tegulum to base of embolus. Embolus thin, curved, tip sharp.

**Female (paratype)**: total length 4.78, carapace 2.03 long, 1.75 wide; abdomen 2.75 long, 2.17 wide. Eye sizes and interdistances: AME 0.14, ALE 0.12, PME 0.09, PLE 0.10; AME–AME 0.07, AME–ALE 0.01, ALE–ALE 0.34, PME–PME 0.11, PME–PLE 0.08, PLE–PLE 0.46, ALE–PLE 0.13. EAW 0.61, CRW 0.82, EAW/CRW 0.74, CRW/CW 0.95. MOA 0.32 long, anterior width 0.31, posterior width 0.30. CH 0.13, CH/AME 1.08. Labium 0.22 long, 0.30 wide. Sternum 1.18 long, 0.96 wide. Leg measurements: I 10.98 (2.52, 0.69, 3.06, 2.13, 2.58), II 8.31 (2.21, 0.66, 2.38, 1.79, 1.27), III 6.67 (1.81, 0.61, 1.46, 1.77, 1.02), IV 10.42 (2.87, 0.67, 2.50, 2.94, 1.44), leg pattern: 1 4 2 3. Spination: femur I pl 5, femur II d 1 pl 5, femur IV d 1, tibia I pv 9 rv 10, tibia II pv 9 rv 8, metatarsus I pv 4 rv 4, metatarsus II pv 4 rv 3.

***Coloration*.** Carapace darker than male, with several patches resembling flowing droplets beside fovea. Abdomen black, with a small, white inverted triangular mark anteriorly. A small, irregular, slightly sclerotized area in front of the spinnerets with black setae. Leg yellow-brown.

***Epigyne*** as in Fig. 4E, F. Epigynal plate slightly sclerotized. Median septum narrow, inverted goblet-shaped. Copulatory openings distinct, separated by median septum. Copulatory ducts thick and strong, bent, J-shaped. Bursa medium, balloon-shaped, the right one larger than left in dorsal view. Glandular appendages large. Connecting tubes short, curved, thinner than copulatory ducts. Spermathecae long, clavate, separated from each other. Fertilization ducts located at posterior of spermathecae.

#### Distribution.

China: Guangdong Province (Fig. 7).

### ﻿ Grandilithus lanxi sp. nov.

Taxon classificationAnimaliaAraneaePhrurolithidae

26A0B53E-57A1-5076-9823-3296950CE297

https://zoobank.org/60B90CE6-543D-4BBD-8868-B2DC66F5354D

**Figure 5.**
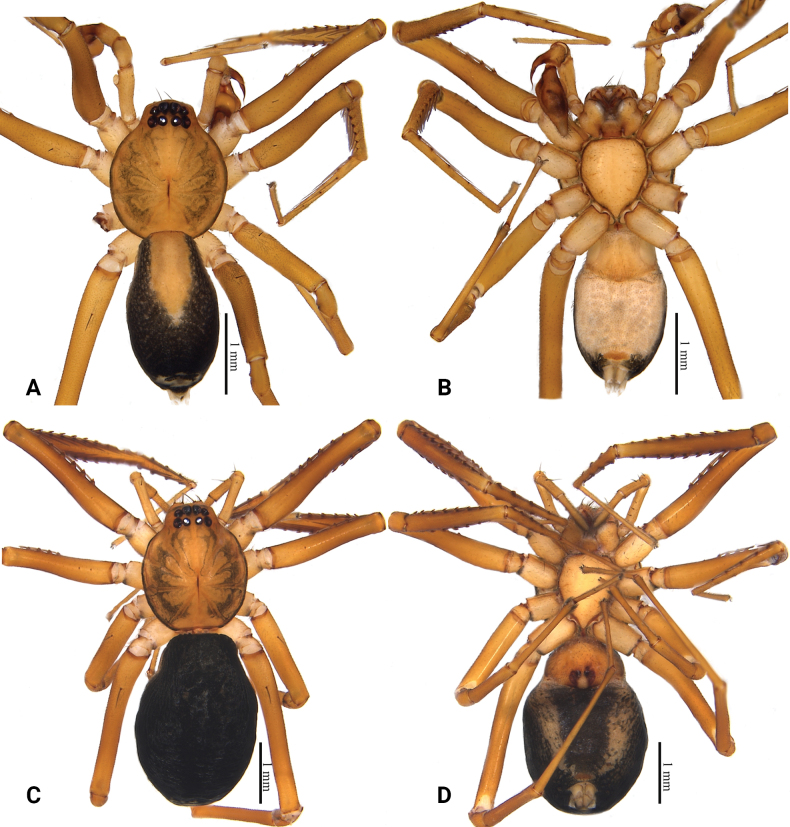
Habitus of *Grandilithuslanxi* sp. nov. **A** male holotype, dorsal view **B** same, ventral view **C** female paratype, dorsal view **D** same, ventral view.

**Figure 6.**
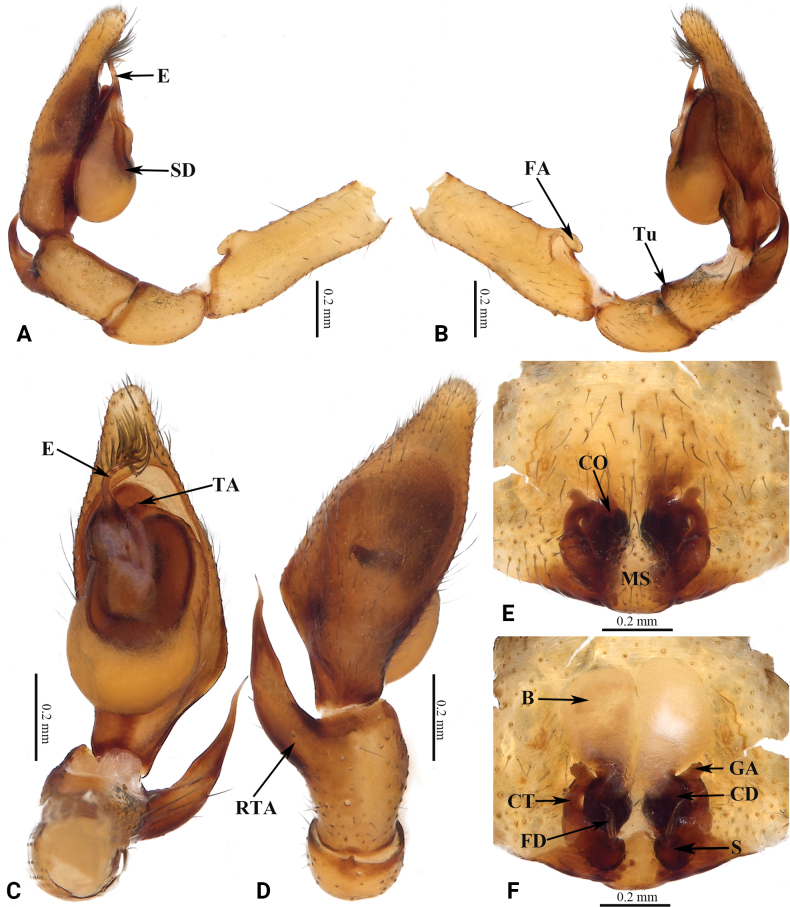
Copulatory organs of *Grandilithuslanxi* sp. nov. **A** male left palp, prolateral view **B** same, retrolateral view **C** same, ventral view **D** same, dorsal view **E** epigyne, ventral view **F** same, dorsal view. Abbreviations: B—bursa; CD—copulatory duct; CO—copulatory opening; CT—connecting tube; E—embolus; FA—femoral apophysis; FD—fertilization duct; GA—glandular appendage; MS—median septum; RTA—retrolateral tibial apophysis; S—spermatheca; SD—sperm duct; TA—tegular apophysis; Tu—tubercle.

Figs 5 , 6 Chinese name: 兰溪大斑蛛

#### Type material.

***Holotype*** ♂ (HBUARA#2021-68): China: Yangmingshan Park, Chongyi County, Ganzhou City, Jiangxi Province, Lanxi Valley (25°39.22'N, 114°18.99'E; 506 m a.s.l.), 28 May 2021, leg. Yannan Mu. ***Paratype*: 3** ♀, with same data as holotype.

#### Etymology.

The specific epithet refers to the type locality.

#### Diagnosis.

This species resembles *G.fengshan* Liu & Li, 2022 in having a similarly shaped embolus and tegular apophysis, but can be recognized by: 1) the long, distally sharp, nearly ensiform-shaped RTA (vs RTA short, tip curved, coracoid-shaped, cf. Fig. 6C, D with fig. 45D–F in Liu et al. 2022); 2) the narrow tegulum (thinner than cymbium) (vs tegulum wider than cymbium, cf. Fig. 6C, D with fig. 45D in Liu et al. 2022); 3) the wide median septum (vs narrower, cf. Fig. 6E, F with fig. 15D, E in Mu and Zhang 2023b), 4) the long, cylindrical glandular appendage (vs short, discoidal, cf. Fig. 6E, F with fig. 15D, E in Mu and Zhang 2023b); and 5) the large spermathecae, connecting tubes thick with broad base (vs connecting tubes thin without broad base, spermathecae small, cf. Fig. 6E, F with fig. 15D, E in Mu and Zhang 2023b).

#### Description.

**Male (holotype)**: total length 3.68, carapace 1.70 long, 1.44 wide; abdomen 1.98 long, 1.12 wide. Eye sizes and interdistances: AME 0.12, ALE 0.10, PME 0.08, PLE 0.09; AME–AME 0.05, AME–ALE 0.01, ALE–ALE 0.29, PME–PME 0.11, PME–PLE 0.06, PLE–PLE 0.38, ALE–PLE 0.08. EAW 0.53, CRW 0.69, EAW/CRW 0.77, CRW/CW 0.48. MOA 0.29 long, anterior width 0.28, posterior width 0.28. CH 0.13. CH/AME 1.08. Labium 0.20 long, 0.25 wide. Sternum 0.96 long, 0.83 wide. Leg measurements: I 8.73 (2.20, 0.63, 2.46, 1.96, 1.48), II 6.79 (1.66, 0.56, 1.84, 1.55, 1.18), III 5.72 (1.44, 0.53, 1.22, 1.60, 0.93), IV 8.86 (2.36, 0.60, 2.04, 2.56, 1.30), leg pattern: 4123. Spination: femur I–IV d 1, femur I pl 5, tibia I pv 8 rv 9, tibia II pv 8 rv 7, metatarsus I pv 4 rv 4, metatarsus II pv 4 rv 2.

***Coloration*.** Carapace yellow-brown, with several patches resembling flowing droplets beside fovea. Abdomen black-gray, with a yellow-brown dorsal scutum longer than 1/2 abdomen length. A small, irregular, slightly sclerotized area in front of the spinnerets with black setae. Leg yellow-brown.

***Palp*** as in Fig. 6A–D. Femoral apophysis protruding. Tubercle distinct. Retrolateral tibial apophysis long, curved, with wide base and sword-like tip. Tegulum oval, slightly thinner than cymbium; tegular apophysis nearly triangular. Sperm duct distinct, tapering from retrolateral of tegulum to base of embolus. Embolus curved, tip blunt.

**Female (paratype)**: total length 4.93, carapace 2.07 long, 1.75 wide; abdomen 2.86 long, 1.95 wide. Eye sizes and interdistances: AME 0.14, ALE 0.12, PME 0.09, PLE 0.10; AME–AME 0.07, AME–ALE 0.01, ALE–ALE 0.35, PME–PME 0.13, PME–PLE 0.09, PLE–PLE 0.48, ALE–PLE 0.09. EAW 0.64, CRW 0.88, EAW/CRW 0.73, CRW/CW 0.50. MOA 0.32 long, anterior width 0.32, posterior width 0.30. CH 0.15, CH/AME 1.07. Labium 0.23 long, 0.32 wide. Sternum 1.25 long, 0.92 wide. Leg measurements: I 9.54 (2.36, 0.76, 2.90, 2.20, 1.32), II 7.68 (1.96, 0.61, 2.17, 1.74, 1.20), III 6.18 (1.65, 0.56, 1.37, 1.67, 0.93), IV 9.63 (2.69, 0.67, 2.21, 2.86, 1.20), leg pattern: 4123. Spination: femur I d 1 pl 6, femur II d 2 pl 4, femur III–IV d 1, tibia I pv 10 rv 10, tibia II pv 9 rv 8, metatarsus I pv 4 rv 4, metatarsus II pv 5 rv 4.

***Coloration*.** Carapace slightly darker than male, with several patches resembling flowing droplets beside fovea. Abdomen black. A small, irregular, slightly sclerotized area in front of the spinnerets with black setae. Leg yellow-brown.

***Epigyne*** as in Fig. 6E, F. Epigynal plate slightly sclerotized. Median septum wide, inverted goblet-shaped. Copulatory openings small, separated by median septum. Copulatory ducts thick. Glandular appendages thick, short-cylindrical. Connecting tubes thinner than copulatory ducts, base broad. Bursa nearly reniform, the right one slightly larger than left in dorsal view. Spermathecae oval, slanted, separated from each other. Fertilization ducts located at posterior of spermathecae.

#### Distribution.

**Figure 7.**
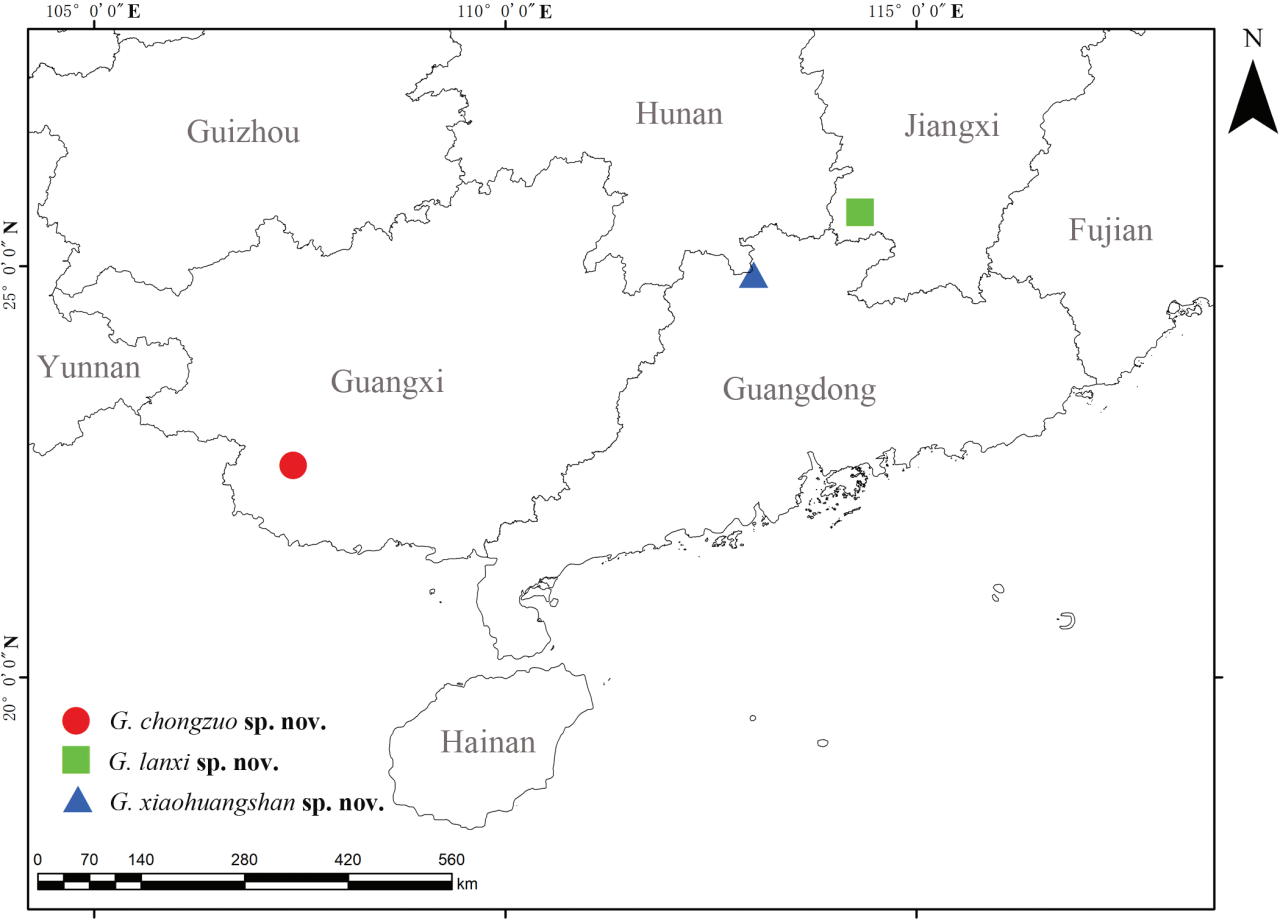
Collection localities of the new *Grandilithus* species described in this paper.

China: Jiangxi Province (Fig. 7).

## Supplementary Material

XML Treatment for Grandilithus

XML Treatment for Grandilithus chongzuo

XML Treatment for Grandilithus xiaohuangshan

XML Treatment for Grandilithus lanxi

## ﻿Additional information

### Author ORCIDs

Mengjiao Xu https://orcid.org/0009-0001-9548-6941

Yannan Mu https://orcid.org/0000-0002-2504-673X

Chao Zhang https://orcid.org/0000-0003-1702-1206

Feng Zhang https://orcid.org/0000-0002-3347-1031

### Data availability

All of the data that support the findings of this study are available in the main text.

## References

[B1] Álvarez-Padilla F Hormiga G (2007) A protocol for digesting internal soft tissues and mounting spiders for scanning electron microscopy. The Journal of Arachnology 35(3): 538–542. 10.1636/Sh06-55.1

[B2] Fu LN He JC Zhang F (2015) Species of the genus *Otacilia* from Hainan Island, China (Araneae: Phrurolithidae). Zoological Systematics 40: 436–450.

[B3] Jin C Li XL Zhang F (2022) First record of the genus *Plynnon* Deeleman-Reinhold, 2001 from China, with the description of a new species (Araneae, Phrurolithidae). Biodiversity Data Journal 10(e85029): 1–8. 10.3897/BDJ.10.e85029 PMC9848520 36761620

[B4] Liu KK Xu X Xiao YH Yin HQ Peng XJ (2019) Six new species of *Otacilia* from southern China (Araneae: Phrurolithidae). Zootaxa 4585(3): 438–458. 10.11646/zootaxa.4585.3.2 31716154

[B5] Liu KK Luo HP Ying YH Xiao YX Xu X Xiao YH (2020) A survey of Phrurolithidae spiders from Jinggang Mountain National Nature Reserve, Jiangxi Province, China. ZooKeys 946: 1–37. 10.3897/zookeys.947.51175 32733127 PMC7363721

[B6] Liu KK Li SQ Zhang XQ Ying YH Meng ZY Fei MH Li WH Xiao YH Xu X (2022) Unknown species from China: The case of phrurolithid spiders (Araneae, Phrurolithidae). Zoological Research 43(3): 352–355. 10.24272/j.issn.2095-8137.2022.055 35355455 PMC9113968

[B7] Mu Y Zhang F (2022) *Lingulatus* gen. nov., a new genus with description of three new species and one new combination (Araneae: Phrurolithidae). Zootaxa 5178(3): 265–277. 10.11646/zootaxa.5178.3.5 36095730

[B8] Mu Y Zhang F (2023a) A new recorded genus and species of phrurolithids in Hebei University of China (Araneae: Phrurolithidae). Journal of Hebei University 43(1): 90–94. 10.3969/j.issn.1000-1565.2023.01.012

[B9] Mu Y Zhang F (2023b) Further additions to the guardstone spider fauna from China (Araneae: Phrurolithidae). Zootaxa 5338(1): 1–104. 10.11646/zootaxa.5338.1.1 38221069

[B10] Mu Y Lin XY Zhang F (2022) First records of the genus *Phrurotimpus* Chamberlin & Ivie, 1935 from China, with two new species and one new combination (Araneae: Phrurolithidae). Zootaxa 5124(5): 565–576. 10.11646/zootaxa.5124.5.5 35391101

[B11] World Spider Catalog (2023) World Spider Catalog. Version 24. Natural History Museum Bern, Bern. 10.24436/2 [Accessed on 23 September 2023]

